# Biocompatible molecularly imprinted polynorepinephrine nanoparticles: rational design and one-step reversible immobilization for enhanced protein recognition by surface plasmon resonance

**DOI:** 10.1007/s00604-026-07893-z

**Published:** 2026-02-19

**Authors:** Simone Ventisette, Giulia Galgani, Pasquale Palladino, Vincenzo Calderone, Valentina Citi, Maria Minunni, Simona Scarano

**Affiliations:** 1 Department of Chemistry “Ugo Schiff’, University of Florence, Via della Lastruccia, 3-13, 50019 Sesto Fiorentino, Italy; 2 Department of Pharmacy, University of Pisa, Via Bonanno 6, 56126 Pisa, Italy

**Keywords:** Molecularly imprinted nanoparticles, Polynorepinephrine, Catecholamines, Biomimetic receptors, Flow-based immobilization, Surface plasmon resonance (SPR)

## Abstract

**Abstract:**

Polynorepinephrine nanoparticles (PNE-NPs) are emerging bioinspired nanomaterials with significant potential in diagnostics and therapy, yet their systematic synthesis and functional assessment remain limited. In this work, a Design of Experiments approach was applied to optimize the synthesis of non-imprinted and imprinted PNE-NPs. A green, pH-triggered precipitation/redispersion protocol was introduced for nanoparticle purification, providing fast and reproducible recovery without organic solvents, and surpassing conventional membrane dialysis methods, which are typically long and labor-intensive. Key parameters (pH, temperature, reaction time, stirring, and monomer concentration) were screened in H_2_O/NaOH and TRIS buffer media. Optimized PNE-NPs displayed hydrodynamic diameters below 200 nm, spherical morphology, and negligible cytotoxicity in HaCaT keratinocytes across a broad concentration range. As a model study, PNE-NPs were imprinted against the Fc portion of human IgG1 and tested as synthetic receptors by surface plasmon resonance (SPR). Two flow-mode immobilization strategies were compared on bare gold chips: covalent grafting on thiol-modified gold and direct adsorption. Both allowed real-time, in-flow monitoring and markedly improved affinity (K_D_ < 10^−8^ mol L^−1^) compared to previous imprinted PNE nanofilms. The adsorption protocol stood out for its simplicity, high affinity and selectivity (α > 27.4), and full in situ reconditioning of the SPR gold transducer with NaClO washes, enabling multiple reuse cycles. These results establish PNE-NPs as versatile synthetic receptors, highlighting their promise as next-generation platforms for diagnostics and therapy.

**Graphical abstract:**

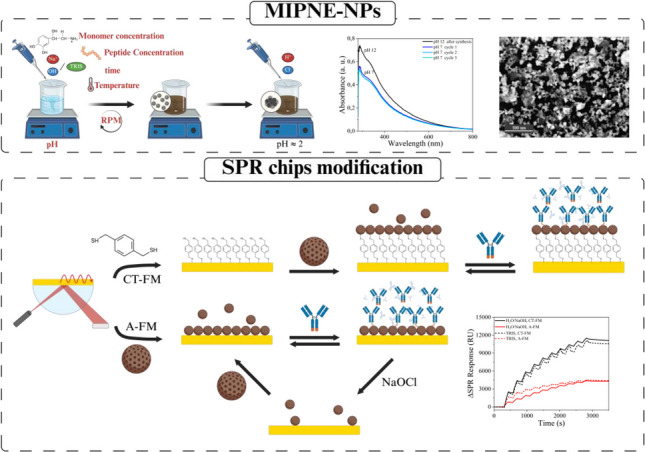

**Supplementary Information:**

The online version contains supplementary material available at 10.1007/s00604-026-07893-z.

## Introduction

Molecularly imprinted polymers (MIPs) are synthetic materials featuring recognition sites complementary in shape, size, and functionality to a target molecule, and are widely recognized as robust alternatives to biological receptors in sensing and separation applications. Owing to their high stability, tunable selectivity, and adaptability to diverse analytes, MIPs have been extensively investigated in bioanalytical contexts. Recent advances have focused on innovative design and fabrication strategies, such as nanoMIP synthesis, rational template selection, and integration with optical and electrochemical transducers, to improve affinity, selectivity, and analytical performance in biomarker detection [1–3]. Within this framework, increasing attention has been devoted to MIP architectures tailored as planar thin films or discrete nanoparticles, as these formats offer distinct advantages in terms of accessibility of recognition sites, integration with transducers, and assay flexibility. In particular, bioinspired polymeric nanoparticles synthesized in aqueous media have emerged as attractive platforms due to their reproducible and environmentally friendly preparation, narrow size distribution, high colloidal stability, and low cytotoxicity [4–7]. When combined with molecular imprinting, such nanoparticles enable the development of multifunctional, biocompatible synthetic receptors suitable for bioanalysis, diagnostics, and related biomedical applications [8–12]. The development of bioinspired polymeric nanoparticles (NPs) has gained growing attention in recent years, particularly in the fields of bioanalysis and therapy, due to their facile and environmentally friendly reproducible synthesis in aqueous media [4, 5], narrow size distribution, high colloidal stability, and low cytotoxicity [6, 7]. Multifunctional NPs combining biocompatibility, fluorescence, cell permeability, and intrinsic biological activity provide versatile platforms for biomedical applications such as bioimaging, diagnostics, and therapy [8–12]. However, achieving such multifunctionality within a single nanoparticle remains challenging, often requiring complex protocols involving size control, surface engineering, and incorporation of fluorophores or therapeutic agents. In this context, the polymerization of catecholamine neurotransmitters such as norepinephrine (NE) has recently emerged as a promising strategy for synthesizing functional NPs [10, 13–15]. Under alkaline conditions, NE undergoes spontaneous oxidative polymerization to yield polynorepinephrine (PNE) NPs. Despite its structural similarity to polydopamine (PDA), PNE contains an additional hydroxyl group that confers distinct physicochemical and biological properties [16, 17]. Although less investigated than PDA, PNE nanoparticles (PNE-NPs) have recently shown substantial potential in bioanalytic and biosensing, particularly in protein detection by molecular imprinting [18–20]. Molecularly Imprinted PNE (MIPNE), previously prepared as adherent nanofilms, has been reported for targeting a range of clinically relevant protein biomarkers. These materials have shown excellent analytical performance, including high selectivity, reproducibility, and low limits of detection (LOD). Applications include bioanalytical assays in microwell plates, such as ELISA-like colorimetric formats (e.g., Biomimetic Enzyme-Linked Assay, BELISA) [18], and optical biosensor platforms, particularly Surface Plasmon Resonance (SPR) [19] and Biolayer Interferometry (BLI) [20]. In these contexts, MIPNE are directly grown as nanofilms on microwell plates or sensor chip surfaces. However, the three-dimensional architecture of the recognition layer dictates both sensitivity and kinetic performance. Compared to planar MIPNE, NPs-based receptors represent a prominent shift towards increased surface-to-volume ratio while interparticle voids create a porous network that shortens diffusion pathways and minimizes steric hindrance. Moreover, spherical geometry ensures that binding sites on all facets remain solvent-accessible, maximizing active site density and accelerating association/dissociation rates. In an explorative study, MIPNE-NPs synthesized in solution demonstrated selective recognition of human IgG1 (hIgG1), both when immobilized as receptors and when injected as analytes against immobilized epitopes [21]. Nonetheless, a systematic investigation of synthetic routes determining the physicochemical features of PNE-NPs-both non-imprinted (NIPNE) and imprinted (MIPNE) is still lacking. To address this gap, a Design of Experiments (DoE) approach was adopted to efficiently explore the multidimensional synthesis space. This statistical method allows simultaneous evaluation of multiple factors and their interactions, providing a deeper understanding of the parameters governing NPs formation. By improving efficiency and reproducibility, DoE enables the fine-tuning of reaction conditions to optimize key features such as size and dispersity-critical for diagnostic and nanomedical applications. In this study, a Plackett-Burman design (PBD) was applied to evaluate five key variables: monomer concentration, reaction temperature, reaction time, stirring speed, and pH, each tested at two levels. The resulting NPs were characterized by Dynamic Light Scattering (DLS), UV-Vis spectroscopy, zeta potential analysis, and electron microscopy (SEM/STEM). Additionally, given the potential of imprinted NPs as emerging nanomaterials for recognizing soluble target analytes [22] and enabling targeted drug delivery and receptor-specific therapies [15], the cytotoxicity of PNE-NPs-both NIPNE and MIPNE-was preliminarily assessed using HaCaT keratinocytes, a well-established model for dermal toxicity and biocompatibility. PNE-NPs display several features advantageous for biomedical use. Their intrinsic fluorescence allows direct imaging without additional labels [14]. They exhibit pH-responsive drug release, enabling targeted delivery in acidic environments such as tumor tissues, thereby improving selectivity and minimizing off-target effects. They also demonstrate high biocompatibility and controllable size, functioning as efficient carriers for chemotherapeutics with minimal cytotoxicity. Different particle sizes and concentrations were evaluated, acknowledging that toxicity may vary depending on surface chemistry, size, and morphology. MIPNE-NPs obtained through this rational approach were subsequently tested as synthetic receptors in real time and label free optical sensing, i.e. Surface Plasmon Resonance (SPR). Two immobilization strategies, covalent/irreversible and non-covalent/reversible, were explored on bare gold chips to identify the optimal balance between analytical performance and simplicity of surface modification. Binding parameters, including both thermodynamic (dissociation constant, K_D_) and kinetic (association rate constant, k_a_, and dissociation rate constant, k_d_) constants, were investigated. As a model system for molecular imprinting, PNE-NPs were tailored to selectively recognize and quantify hIgG1. The optimization of the selected epitope is grounded in previous systematic studies in which different epitopes located in distinct regions of the hIgG molecule were comparatively evaluated [23]. Among them, the C-terminal epitope ^441^KSLSLSPGK^449^ was identified as the most effective, exhibiting high selectivity toward the target hIgG, subclass IgG1. These results demonstrated that epitope selection is a critical determinant of MIPNE performance, particularly in terms of target specificity. Building on this evidence, the same epitope was subsequently adopted in further investigations, where MIPNE nanofilms were developed and successfully integrated with different optical transduction platforms, including SPR [21, 24] and BLI [20]. This work therefore provides a comprehensive investigation of both synthetic optimization and functional performance of PNE-NPs, from non-imprinted to molecularly imprinted formats. By combining DoE-guided synthesis with systematic binding studies, we demonstrate that MIPNE-NPs outperform conventional nanofilms, offering higher binding strength, superior specificity, and reusable immobilization strategies under flow. These advances highlight the potential of MIPNE-NPs as next-generation synthetic receptors for diagnostic biosensing and possibly open a perspective for in vivo studies in therapeutic applications.

## Materials and methods

### Synthesis of non-imprinted PNE-NPs

Non-imprinted PNE-NPs (NIPNE-NPs) with hydrodynamic diameters (D_h_) of 100–400 nm were synthesized via a one-pot, non-covalent polymerization-induced precipitation method. Process optimization was performed using a PBD with Desice® software (Rupert Brandstätter, Scheffau am Tennengebirge, Austria). Two synthesis media were investigated: H_2_O/NaOH and 10 mmol L^−1^ TRIS buffer. The experimental matrices and factor levels are reported in Table S1, and the complete experimental plans in Tables S2-3. For H_2_O/NaOH syntheses, 2 mL of NE solution (1–5 g L^−1^; MW 205.64 g mol^−1^) in Milli-Q water was stirred at 400–1000 rpm for 5 min at 30–70 °C. Then, 1 mol L^−1^ NaOH was added to adjust pH (11.0–13.0). Polymerizations proceeded for 2–8 h, after which NP-enriched pellets were obtained by acidification to pH < 2 with 200 μL of 1 mol L^−1^ HCl. The TRIS protocol followed the same steps, with NE dissolved directly in a 10 mmol L^−1^ TRIS buffer at pH 7.0–11.0. Reactions were incubated at 30–70 °C under stirring (400–1000 rpm) for 2–8 h, and pellets collected as above. In both cases, NPs were purified by three cycles of centrifugation (14,500 g, 10 min), sonication (5 min), and resuspension in 1 mL Milli-Q water, until clear supernatants confirmed removal of unreacted monomers and oligomers. Final pellets were stored at 4 °C until use.

### Synthesis of molecularly imprinted PNE-NPs for detection of hIgG1

Molecularly imprinted PNE-NPs (MIPNE-NPs) were synthesized using the same procedure as for NIPNE-NPs, but in the presence of the small peptide ^441^KSLSLSPGK^449^ (UniProt ID: P0DOX5, IGG1_HUMAN), used as the epitope template. The peptide was introduced at a concentration of 200 μmol L^−1^ in H_2_O/NaOH or 100 μmol L^−1^ in the TRIS buffer protocol, respectively. Selected from the Fc constant region of hIgG1, this peptide was identified through analysis of the amino acid sequence and the X-ray crystal structure of the hIgG1 heavy chain (UniProt P0DOX5) [23]. Molecular imprinting was performed by incorporating the peptide directly at the beginning of the reaction. The resulting MIPNE-NPs were recovered by centrifugation (14,500 g, 10 min), followed by washing with 1 mL of water. The pellet was resuspended in 1 mL of a solution containing 1 mmol L^−1^ 6-mercapto-1-hexanol (MCH) and 1 mmol L^−1^ 11-mercapto-1-undecanol (MCU) in an EtOH:H_2_O mixture (10:90, v/v). The suspension was incubated overnight at 25 °C under magnetic stirring (700 rpm) to passivate the NPs surface. After incubation, MIPNE-NPs were washed with a 20:80 (v/v) EtOH:H_2_O mixture to remove excess thiol compounds. To eliminate any unreacted monomers and residual peptides embedded in the NPs surfaces, four washing cycles were carried out using ACN:H_2_O (10:90, v/v) followed by Milli-Q water. The final pellets were stored at 4 °C until utilization.

### PNE-NPs characterization

#### UV-vis spectroscopy

UV-Vis measurements were performed on a SPECTROstar Nano® microplate absorbance reader (BMG Labtech, Ortenberg, Germany) within the range 285–800 nm, at room temperature.

#### Dynamic light scattering (DLS) and zeta potential (Zpot)

Dynamic Light Scattering measurements (size and Z-potential) were performed on 200 μl of NPs suspension using a Zetasizer Pro® instrument (Malvern Panalytical, Malvern, UK). To minimize multiple scattering effects due to the high particle concentration, the samples were properly diluted in Milli-Q water prior to analysis.

#### Scanning Electron microscopy (SEM) and scanning transmission Electron microscopy (STEM)

SEM images were acquired with a Phenom Pharos G2 FEG-SEM (Thermo Fisher Scientific, Waltham, MA, USA) on dried 4 μL MIPNE-NPs drops (graphite-coated, 3 nm) under high vacuum (0.10 Pa, 20 kV), while STEM analyses were performed on Formvar-Carbon copper grids at 15 kV using the SEM-integrated STEM detector. NP diameters were measured from at least 200 particles per sample using Phenom Pro Suite particlemetric software (see Supporting Information for details).

#### Surface Plasmon resonance (SPR)

Biacore X100™ system (Cytiva AB, Uppsala, Sweden) was used to monitor PNE-NPs immobilization and to evaluate PNE-MIPs binding performance.

### SPR chips modification with PNE-NPs

**Fig. 1:**
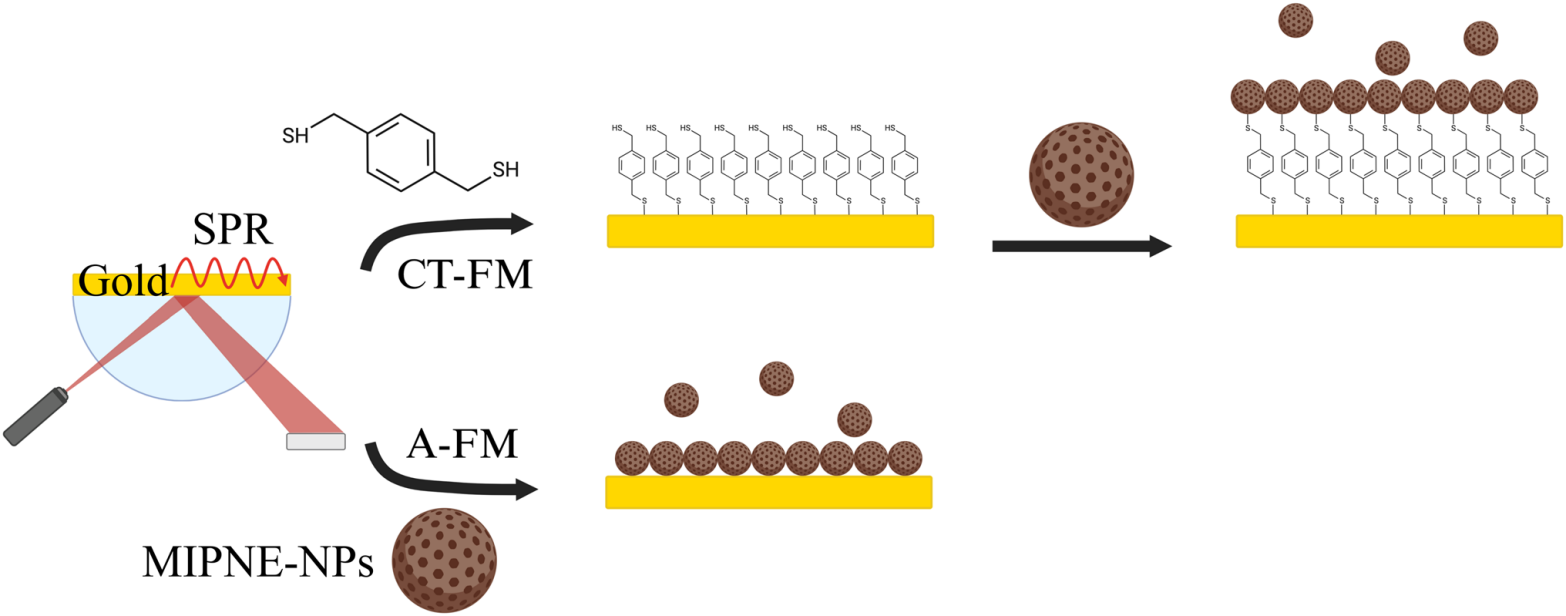
Schematic representation of PNE-NPs immobilization on SPR gold chips under flow mode (FM) via: (i) CT-FM, covalent immobilization through a two-step workflow involving the formation of a thiol self-assembled monolayer followed by covalent linking of MIPNE-NPs via Michael addition; and (ii) A-FM, adsorption-based immobilization of MIPNE-NPs directly on bare gold, exploiting the intrinsic adhesive properties of PNE

Two immobilization strategies were investigated for anchoring of MIPNE-NPs onto SPR sensor surfaces (Fig. 1). Gold sensor chips (SIA Au kit, Cytiva AB, Uppsala, Sweden) were employed as substrates. Immobilizations were performed under flow mode (FM). As a reference, gold chips modified with MIPNE nanofilms were employed (paragraph 2.4.3).

#### Covalent immobilization on thiol-modified gold chips (CT-FM)

A 200 μL solution of 1 mmol L^−1^ 1,4-benzenedimethanthiol (BDMT) in Milli-Q water was drop-cast onto bare gold chips to form a self-assembled monolayer. Chips were incubated for 20 h at room temperature in the dark, in a humidified chamber under vacuum. Surfaces were then rinsed with Milli-Q water and an EtOH:H_2_O (20:80, v/v) mixture to remove excess reagents. Chips were mounted in the instrument and functionalized via Michael addition with MIPNE-NPs (reconstituted in 350 μL PBS) [25]. Immobilization was performed under flow with 10 sequential injections (contact time: 120 s each) [21]. A final conditioning step (two 30 s serial injections of 10 mmol L^−1^ HCl and 0.005% (v/v) SDS) was applied before baseline stabilization and characterization.

#### Adsorption on bare gold chips (A-FM)

For adsorption (A-FM), gold chips were directly functionalized by spontaneous physisorption of MIPNE-NPs (reconstituted in 350 μL PBS) under flow. Ten sequential injections were performed (120 s each). Conditioning involved two 30 s injections of 10 mmol L^−1^ HCl and 0.05% (v/v) SDS, followed by one 30 s injection of 10 mmol L^−1^ HCl. Baseline stabilization (1500 s) preceded characterization. After each measurement set, chip surfaces were reconditioned under flow with 0.1 mol L^−1^ NaOCl, following a reported protocol [26], and reused.

#### MIPNE-film synthesis

MIPNE-based films were directly formed on bare gold SPR chips as previously described by Sestaioni et al. [26]. A 10 mmol L^−1^ TRIS solution (pH 8.50) containing 2.00 g L^−1^ (11.82 mmol L^−1^) NE and 400 μmol L^−1^ of the peptide template was dropped and incubated at 25.0 ± 0.5 °C for 5 h avoiding evaporation. Surfaces were then passivated with 10% EtOH in water containing 1 mmol L^−1^ MCH and 1 mmol L^−1^ MCU. The peptide template was removed by washing three times with 1 mL of 5% (v/v) CH_3_COOH, and the surface neutralized with 2 mL Milli-Q water. Conditioning was performed after chip mounting in the SPR system: six 30 s injections of 5% (v/v) CH_3_COOH, followed by two 30 s injections of 10 mmol L^−1^ HCl and 0.005% (v/v) SDS, prior to baseline stabilization (2000 s) and analysis.

### MIPNE-NPs modified chips: hIgG1 binding performances and calibration by SPR analysis

Kinetic and affinity parameters were determined by SPR, using the Single Cycle Kinetics (SCK) Wizard. A two-state model was applied for peptide binding and a 1:1 L model for protein interactions. Data were processed with BIAevaluation 3.1 software (Cytiva AB). Unless otherwise stated, measurements were performed at 25.0 ± 0.5 °C and 5.00 μL min^−1^ flow rate. Each SCK cycle consisted of five sequential injections of increasing analyte concentrations, with 120 s association and 60 s dissociation phases, without intermediate regeneration. For peptide-binding studies, concentration ranged from 3.75 to 60 μmol L^−1^ in PBS (pH 7.4). After each SCK cycle, surfaces were regenerated with two 30 s injections of 0.05% (v/v) SDS and 10 mmol L^−1^ HCl. Binding to the target peptide (^441^KSLSLSPGK^449^) was compared to a negative control peptide (^338^ISKAKGQP^345^), also derived from the hIgG1 heavy chain constant domain. For protein analysis, hIgG1 was injected at 13.3–53.3 μmol L^−1^ in the HBS-EP buffer (pH 7.4). Surfaces were regenerated with a single 30 s injection of 10 mmol L^−1^ NaOH. All experiments were performed in triplicate. Specificity was further assessed against other human serum immunoglobulins, following Ventisette et al. [20].

### MIPNE-NPs modified chips: Calibration of hIgG1 in buffer and serum

Human serum was used as a representative complex matrix. hIgG1 was spiked (13.3–53.3 μmol L^−1^), diluted 2000-fold in HBS-EP buffer (pH 7.4) and filtered through Amicon® Ultra centrifugal filters (100 kDa) to remove endogenous immunoglobulins prior to SPR analysis. Multi-cycle kinetics (MCK) experiments were performed in triplicate for calibration in buffer and serum, using five sequential injections of increasing analyte concentrations, a dissociation phase of 600 s, and surface regeneration with two 30 s injections of 10 mmol L^−1^ NaOH.

### Evaluation of NIPNE-NPs and MIPNE-NPs toxicity using HaCat cells

Human immortalized keratinocytes (HaCaT) were cultured in Dulbecco’s Modified Eagle’s Medium-High Glucose (DMEM-HG) supplemented with 10% fetal bovine serum (FBS) and 1% penicillin-streptomycin (10,000 U mL^−1^ penicillin, 10 mg mL^−1^ streptomycin). Cells were maintained at 37 °C in a humidified incubator with 5% CO_2_. At confluence, HaCaT were seeded at 10,000 cells per well in 96-well plates and incubated overnight. Lyophilized NIPNE- and MIPNE-NPs were reconstituted in Dulbecco’s PBS (1 mg mL^−1^) and sonicated for 10 min to ensure dispersion. Cells were treated for 24 h with NPs suspensions at 10, 30, and 100 ng mL^−1^ in culture medium, each at least in triplicate. Cell viability was assessed using the water-soluble tetrazolium-1 (WST-1) assay, which quantifies mitochondrial succinate dehydrogenase activity in metabolically active cells through the reduction of tetrazolium salts to soluble formazan. The WST-1 assay was selected for its high sensitivity, allowing accurate viability assessment without impairing cellular integrity. Importantly, the use of this assay is consistent with the OECD *Guidance Document on Good* In Vitro *Method Practices* (GIVIMP), which recommends the application of well-characterized, reproducible, and non-destructive assays for reliable in vitro toxicity and viability evaluation. After a 1 h incubation at 37 °C in a humidified atmosphere containing 5% CO₂, absorbance was measured at 450 nm using an EnSpire® multimode spectrophotometer. (PerkinElmer, USA). Data were analyzed with GraphPad Prism.

## Results and discussion

### Development of a pH-triggered purification protocol for PNE-NPs

**Fig. 2:**
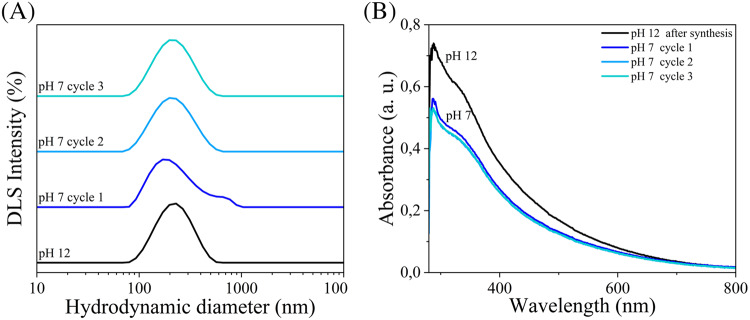
pH-triggered precipitation-based purification of PNE-NPs. **A** Hydrodynamic diameter (D_h_) distribution and **B** UV-Vis absorption spectra collected for three sequential cycles of acid-induced precipitation (pH 1), washing, and redispersion at neutral pH (pH 7)

As a first step, we developed a novel, aqueous-based purification method for PNE-NPs that is simple, sustainable, and overcomes key limitations of conventional protocols. Existing procedures typically rely on organic solvents and ultracentrifugation or dialysis steps, which are both time-consuming and environmentally burdensome [14, 15, 23, 27]. Building on a previously published synthesis protocol [21], the method exploits the colloidal behavior of PNE-NPs as a function of pH. At neutral pH, PNE-NPs exhibit a negative surface potential (≈ −35 mV), which is consistent with good colloidal stability. Upon acidification by addition of 1 mol L^−1^ HCl, protonation of surface functional groups leads to a progressive reduction of electrostatic repulsion between particles (≈ 0 mV at pH 2), resulting in efficient aggregation and precipitation (Fig. S1). This pH-responsive behavior is consistent with the catecholamine nature of PNE and the pH-dependent ionization of its catechol and amine groups. Inspired by this spontaneous behavior, we explored the reversibility of this process to simplify, improve, and speed up the purification and collection of these nanomaterials after synthesis. To this aim, several pH-triggered precipitations, washing and redispersion cycles were performed, checking by DLS, zeta potential, and UV-Vis measurement the efficiency of the process. Therefore, after NPs acidification, the pellet was collected by centrifugation (14,500 rpm, 10 min) and then redispersed by simply readjusting the pH to neutrality. As reported in Fig. 2, the procedure allowed for a reproducible and efficient cycle of purification. To our knowledge, no comparable pH-triggered protocol has been reported for PNE-NPs, making this approach a significant advancement toward faster, greener, and more accessible processing. This strategy represents a crucial step toward enabling broader application of PNE-based nanomaterials in diagnostics and nanomedicine, where reproducibility and scalability are essential requirements. The sustainability of the approach is fully consistent with recent advances in molecular imprinting [28]. As highlighted, the use of biopolymer building blocks synthesized directly in water represents a key step toward greener, biocompatible imprinting strategies. By eliminating auxiliary reagents and relying on solvent-free or aqueous conditions, the developed MIPNE-NPs align with the broader trend toward sustainable and bio-based affinity materials.

### Design of experiments (DoE) to explore NIPNE-NPs synthesis formulations

DoE optimization was performed in parallel using two independent Plackett-Burman matrices: one in H_2_O/NaOH and one in 10 mmol L^−1^ TRIS buffer, to compare their influence on NE polymerization. Although both media provide the alkaline conditions necessary for NE auto-oxidation, which is strongly pH-dependent, their buffering capacities and chemical interactions are substantially different. The factor levels (Tables S2-3) were defined based on prior knowledge of PNE polymer growth and literature reports [10, 14, 15, 21, 27, 29–31]. pH values reflected the typical alkaline range for spontaneous polymerization (11–13 in H_2_O/NaOH; 7–11 in TRIS). Temperatures (30–70 °C) were varied to include mild and accelerated regimes, as higher values enhance kinetics and influence morphology. Reaction times (2–8 h) were tested to evaluate effects on particle growth and uniformity. Stirring speed (400–1000 rpm) was included for its influence on size and dispersity. NE concentrations (1–5 g L^−1^) were chosen to probe the role of monomer availability on NPs yield, in line with recent reports on PNE and other catecholamine-derived nanomaterials [15].

NIPNE-NPs obtained across the design space were characterized by DLS (D_h_, polydispersity index-PDI) and UV-Vis spectroscopy. Absorbance at 700 nm was monitored to avoid overlap with the intrinsic bands of NE and its oxidation products (≈250, 279, 301 nm), ensuring that the signal reflected NP-associated scattering rather than molecular absorption [32]. This wavelength thus provided a synthesis-independent metric of optical density across experiments. Both NaOH- and TRIS-based syntheses yielded NIPNE-NPs with similar average sizes (309 and 348 nm, respectively) but different distributions (Table S4). Particles from H_2_O/NaOH showed a slightly broader size range (126–721 nm) compared to TRIS (170–700 nm). Formulations 1_TRIS and 2_TRIS did not allow NPs formation, indicating that the combination of the lowest temperature (30 °C) and neutral pH (7.00) impaired the polymerization process. Instead, all the syntheses in NaOH yielded to valuable PNE-NPs. However, when considering yields in terms of absorbance at 700 nm, several formulations resulted in low product recovery. Only 2_NaOH and 6/8/9_NaOH (triplicates) reached an optical density > 0.10. Among TRIS formulations, only 5/6/11_TRIS (triplicates) and 8_TRIS achieved comparable values, with 8_TRIS showing the highest yield (1.095 ± 0.003 a.u.), likely due to simultaneous application of maximal factor levels. Average PDIs were consistent (0.27 in NaOH, 0.31 in TRIS), though broader distributions were observed in TRIS syntheses. Considering diagnostic and nanomedical applications, where D_h_ < 200 nm is favorable for microfluidic integration (e.g., SPR biosensing) and systemic delivery, conditions were further refined to minimize NP size. In NaOH syntheses, 2_NaOH exhibited the smallest D_h_ and good yield but required extreme conditions. The 6/8/11_NaOH formulation (pH 12, 50 °C, 5 h, 700 rpm, 3 g L^−1^ NE) was therefore selected and optimized. Lowering NE concentration to 2 g L^−1^ yielded particles with D_h_ 180 ± 2 nm, PDI 0.14 ± 0.02, and optical density 0.209 ± 0.001 a.u. In TRIS syntheses, high-yield formulations (5/6/11_TRIS and 8_TRIS) produced large NPs. Despite optimization attempts, size reduction with acceptable PDI and yield was not achieved. Thus, formulation 3_TRIS was selected: although slightly below the yield threshold (0.10 a.u.), it produced NPs with D_h_ 170 ± 1 nm.

**Fig. 3:**
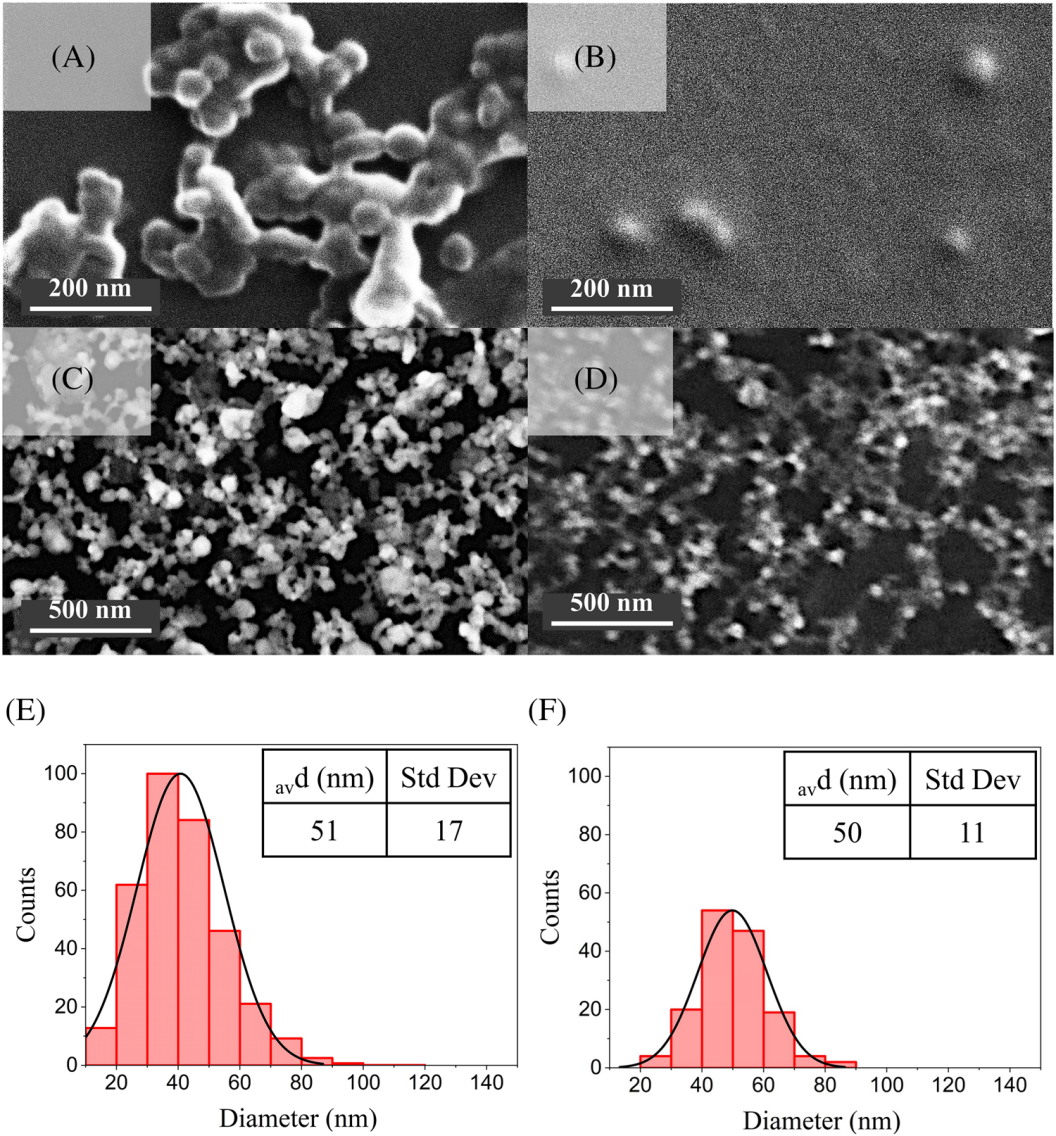
Morphological characterization and size distributions of NIPNE-NPs synthesized in H_2_O/NaOH (A-C-E) versus TRIS (B-D-F) buffer. **A**-**B** SEM images; **C**-**D** STEM images; **E**-**F** Distribution of NIPNE-NPs diameters in STEM images

SEM and STEM further confirmed spherical morphology and unimodal core size distributions, with average dry diameters of 51 ± 17 nm (H_2_O/NaOH) and 50 ± 11 nm (TRIS) (Fig. 3). The larger D_h_ measured by DLS compared to EM are consistent with the hydrated polymer corona surrounding each NP in aqueous suspensions.

### In vitro toxicity of the NIPNE-NPs on human cell cultures

The cytotoxicity of NIPNE-NPs was preliminary assayed in HaCaT keratinocytes, a widely used model for dermal toxicity owing to their physiological relevance to human epidermis. Cells were treated with NPs synthesized in TRIS (3_TRIS, 8_TRIS) and H_2_O/NaOH (2_NaOH, 8_NaOH, optimized 3_NaOH), at concentrations of 10, 30, and 100 ng mL^−1^. Across all formulations and doses, cell viability was comparable to vehicle controls. No significant reduction was observed even at 100 ng mL^−1^, confirming a favourable biocompatibility profile and no cytotoxic effects attributable to the synthesis medium (Fig. S2), in agreement with previous reports [14]. Comparison of NPs synthesized within the same medium but differing in size (125.9 ± 0.7 to 246 ± 6 nm for H_2_O/NaOH; 170 ± 1 to 294 ± 3 nm for TRIS) revealed no correlation between particle diameter and cytotoxicity, with all groups maintaining viability levels indistinguishable from controls. Notably, batch 8_NaOH showed the highest mean viability values at 30 and 100 ng mL^−1^. Although not statistically significant, this trend may indicate a mild proliferative or cytoprotective effect associated with this specific formulation and size range.

### MIPNE-NPs immobilization methods for hIgG1 SPR biosensing

**Fig. 4:**
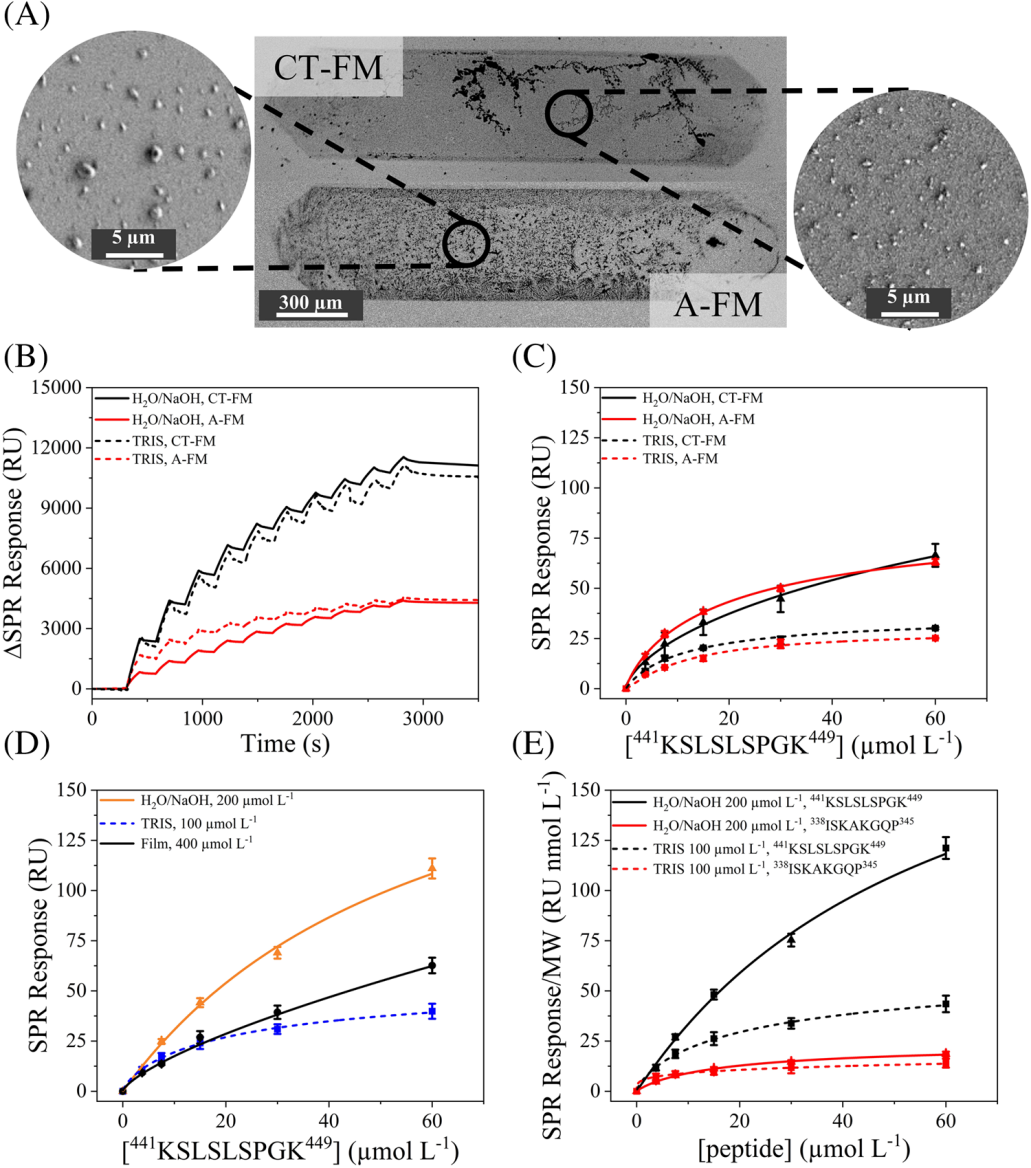
**A** SEM characterization of CT-FM/A-FM modified gold chip; **B** immobilization levels obtained by using CT-FM and A-FM for MIPNE-NPs; **C** SCK analysis of the hIgG1 epitope (^441^KSLSLSPGK^449^, 3.75–60 μM in PBS, pH 7.4) on MIPNE-NPs immobilized via A-FM (red) and CT-FM (black); **D** comparison of MIPNE-NPs with a reference MIPNE nanofilm; (E) selectivity of the MIPNE-NPs prepared in H_2_O/NaOH versus a non-specific peptide (^338^ISKAKGQP^345^)

In our previously developed method [26] MIPNE nanofilms were obtained by drop-casting the monomer-template mixture directly onto the chip surface and allowing polymer growth for 5 h. While versatile, this method does not allow accurate quantification of immobilized receptor material. In contrast, by NPs in situ immobilization under real-time monitoring, it is possible to precise control of surface loading through sequential injections. Moreover, the NPs immobilization can be completed within a few minutes, providing a substantial gain in speed and reproducibility compared to film-based methods. The influence of the hIgG1 epitope concentration on NP imprinting, colloidal properties, and binding performance was systematically evaluated and is discussed in detail in the Supporting Information (Sections S3 and S4). Briefly, modulation of the initial template concentration affected both NP growth and imprinting efficiency in a synthesis-medium-dependent manner, resulting in distinct trade-offs between binding capacity and affinity. Based on this optimization, MIPNE-NPs synthesized in H_2_O/NaOH using an epitope concentration of 200 μmol L^−1^ provided the best overall compromise between high R_max_ and low K_D_ and were therefore selected for subsequent immobilization and biosensing experiments. To implement the NP-based strategy, we further explored two approaches on bare gold chips: (i) covalent immobilization on thiol-functionalized surfaces (CT) and (ii) direct adsorption on bare gold (A), both performed under flow mode (FM) (Fig. 4A). In CT-FM, MIPNE-NPs were covalently attached to a BDMT self-assembled monolayer (SAM) via Michael addition [25] between thiols and the β-carbon of quinone moieties in the PNE matrix. This required a preliminary SAM formation step, which can be performed off instrument. Once the SAM was established, MIPNE-NPs were immobilized under flow through a ten-step injection sequence (Fig. 4B), as previously described by [21]. In the second protocol (A-FM), the direct MIPNE-NPs adsorption onto bare gold surfaces was achieved upon contact during injection, while maintaining the ten-step injection sequence for comparison (Fig. 4B). CT-FM yielded a significantly higher immobilization signal (≈11,000 ∆RU) than A-FM (≈4000 ∆RU). The thiol SAM likely provided a more ordered anchoring scaffold, enabling denser and more uniform NP attachment, enhancing the local refractive index contrast. In A-FM, the absence of a guiding layer led to less efficient, heterogeneous NP distribution and weaker optical response. Notably, this difference appears to be independent of the synthetic route used to obtain the nanoparticles. However, kinetic analysis (Fig. 4C, Table S5) revealed that the synthesis medium plays a dominant role in determining binding behavior: H_2_O/NaOH-prepared MIPNE-NPs exhibited comparable binding levels across both immobilization methods, indicating a generally higher affinity and binding capacity independent of surface anchoring strategy. For particles synthesized in TRIS, CT-FM yielded moderate affinity (K_D_ ≈ 1 × 10^−5^ mol L^−1^) and an R_max_ of ≈ 35 RU, while A-FM resulted in significantly stronger binding (K_D_ ≈ 1 × 10^−8^ mol L^−1^) with a similar R_max_ (≈ 34 RU). NPs synthesized in H_2_O/NaOH showed superior performance overall, with nearly double the binding capacity (R_max_ ≈ 79 RU) across both immobilization methods. Notably, A-FM provided the highest affinity observed (K_D_ ≈ 4 × 10^−10^ mol L^−1^), while maintaining an R_max_ comparable to CT-FM. Beyond delivering strong binding interactions, A-FM also avoids irreversible attachment, allowing sensor surface regeneration and enhancing reusability. Benchmarking MIPNE-NPs against a MIPNE nanofilm showed that, although the film reached the highest R_max_ (≈ 108 RU), its affinity (K_D_ ≈ 3 × 10^−5^ mol L^−1^) was much weaker than the best-performing NPs, underscoring the superior binding strength of NPs-based receptors (Fig. 4D). Finally, sensor specificity was assessed using a non-target peptide (^338^ISKAKGQP^345^), derived from the same constant region of the hIgG1 heavy chain, showing negligible binding (Fig. 4E). The resulting average selectivity factor, defined as α = S_target_/S_interferen_, was 6.53 ± 0.08 for the NaOH-based NPs and 2.5 ± 0.6 for the TRIS-based NPs, confirming the specific recognition of the imprinted epitope and demonstrating superior specificity for the H_2_O/NaOH-derived formulation.

### Morphological and biocompatibility characterization of MIPNE-NPs

**Fig. 5:**
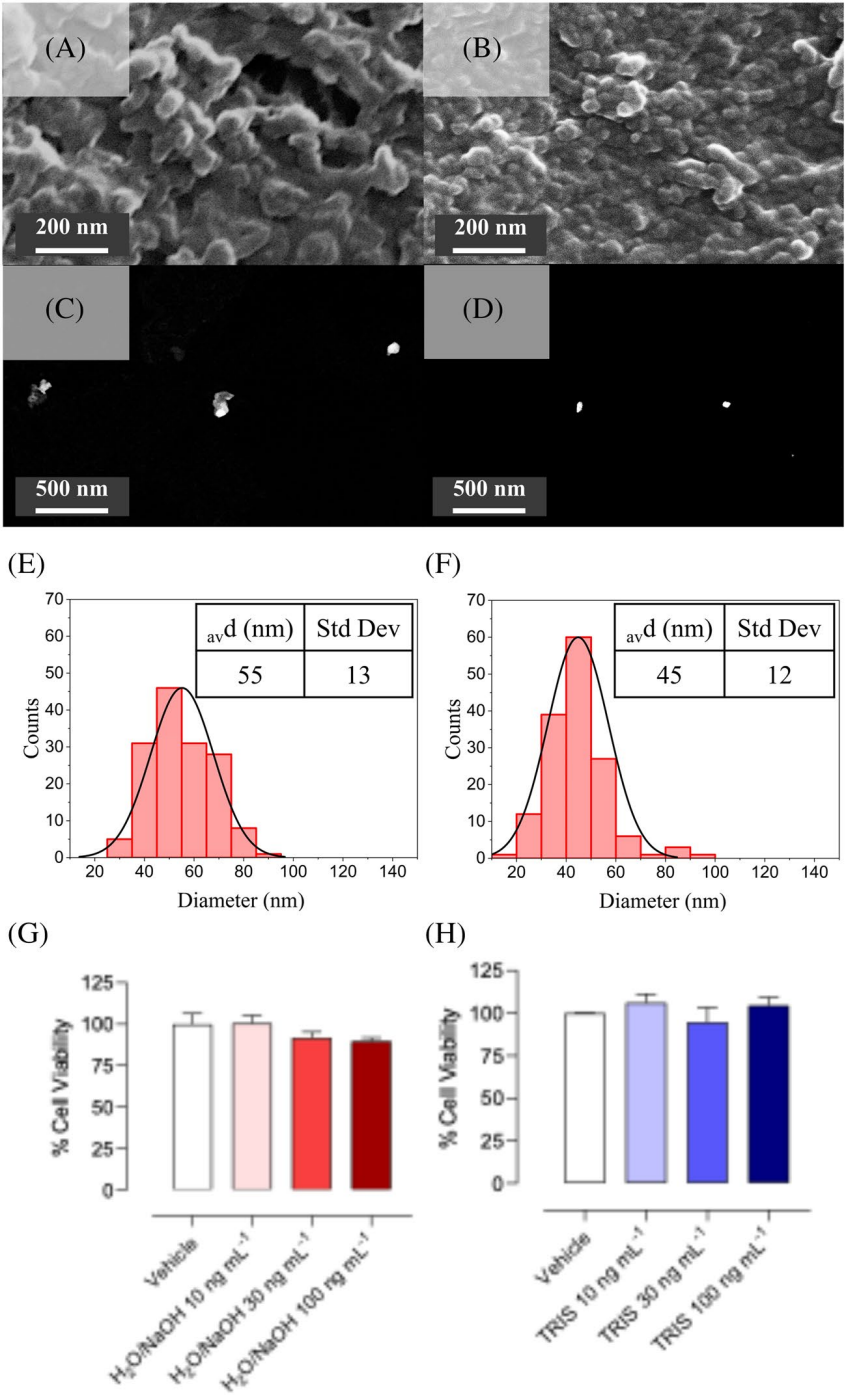
Morphological characterization and size distributions of MIPNE-NPs synthesized in H_2_O/NaOH at 200 μmol L^−1^ epitope (**A**-**C**-**E**-**G**) versus TRIS at 100 μmol L^−1^ epitope (**B**-**D**-**F**-**H**) buffer. **A**-**B** SEM images; **C**-**D** STEM images; **E**-**F** Distribution of MIPNE-NPs diameters in STEM images; (**G**-**H**) cell viability

The selected MIPNE-NPs (H_2_O/NaOH at 200 μmol L^−1^ and TRIS at 100 μmol L^−1^ epitope concentrations) were further characterized to define their morphological features and biocompatibility. SEM and STEM analyses confirmed a spherical morphology and unimodal core size distributions, which were preserved when transitioning from NIPNE to MIPNE NPs, with average dry diameters of 55 ± 13 nm (H_2_O/NaOH at 200 μmol L^−1^ epitope) and 45 ± 12 nm (TRIS at 100 μmol L^−1^ epitope), respectively (Fig. 5A-F). The cytotoxicity of these MIPNE-NPs was then assessed on HaCaT keratinocytes. Overall, the NPs exhibited negligible cytotoxic effects at the tested concentrations, indicating that the molecular imprinting process does not compromise NPs biocompatibility (Fig. 5G-H).

### hIgG1 binding on MIPNE-NPs

#### Kinetic analysis in buffer condition

**Fig. 6:**
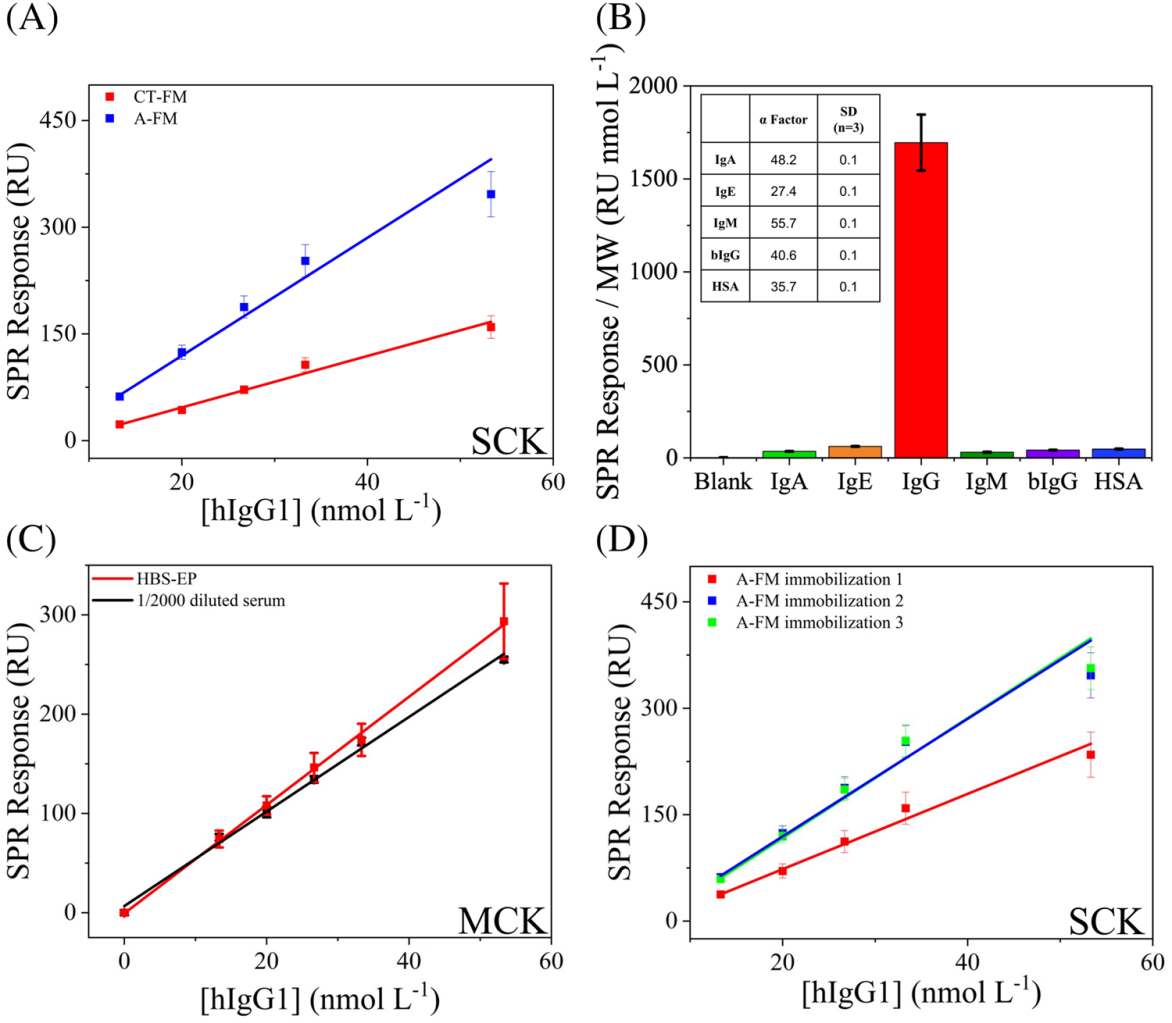
**A** Accumulation points from SCK analysis of hIgG1 using MIPNE-NPs synthesized in H_2_O/NaOH (200 μmol L^−1^ epitope), immobilized via CT-FM (red) and A-FM (blue). **B** Specificity test of the MIPNE-NPs against IgA, IgE, IgM, bIgG, and HSA in HBS-EP, normalized by protein molecular weight (*1000). Selectivity factors (α) relative to hIgG1 are shown in the inset. **C** MCK calibration of hIgG1 in buffer (red) and in human serum (black). **D** Accumulation points from SCK analysis of hIgG1over three consecutive immobilization cycles via A-FM

To evaluate the binding performance of MIPNE-NPs towards the full-length protein, standard hIgG1 was tested in buffer solution (13.3–53.3 nmol L^−1^, HBS-EP buffer, pH 7.4) via SCK analysis. The recognition of hIgG1 by MIPNE-NPs relies on an affinity-based mechanism typical of epitope-imprinted polymers, where selective binding arises from the combined effects of shape complementarity and multiple weak, cooperative interactions. During polymerization, the imprinting epitope guides the local organization of the PNE network, leading to the formation of binding sites capable of selectively recognizing the corresponding region within the native protein. Electrostatic interactions, hydrogen bonding, and hydrophobic contributions are expected to jointly stabilize the complex, consistent with widely accepted models for protein-MIP recognition. MIPNE-NPs synthesized in H_2_O/NaOH were tested using both covalent and adsorption immobilization. Among these, the CT-FM provided the highest binding capacity, with R_max_ values of (4.3 ± 0.3) × 10^3^ RU and a very low K_D_ ((2.2 ± 0.7) × 10^−10^ mol L^−1^), highlighting the high affinity of this strategy but at the cost of a more elaborate surface chemistry (Table S8). The A-FM, while yielding lower R_max_ (844 ± 8 RU), still achieved nanomolar affinity (K_D_ = (1.53 ± 0.03) × 10^−8^ mol L^−1^), and did so through a much simpler and easily regenerable immobilization scheme. By contrast, the direct use of a MIPNE thin film resulted in much lower binding capacity (R_max_ = 108 ± 1 RU) and markedly weaker affinity (K_D_ = (4.8 ± 0.1) × 10^−5^ mol L^−1^). The analysis of the responses extracted from SCK, plotted as a function of protein concentration, further supported these findings (Fig. 6A). While this outcome suggests a format-dependent difference in receptor performance for the selected system, the present data do not allow drawing generalized mechanistic conclusions on the relative merits of nanoparticle- versus film-based MIPNE architectures. At this stage, differences in the effective accessibility of imprinted sites represent a plausible interpretation for the observed behavior, without implying a universal trend across different imprinting systems.

#### hIgG1 determination in buffer and human serum

First, the receptor specificity was assayed against structurally related and unrelated proteins, including human IgA (≈ 160 kDa), IgE (≈ 190 kDa), IgM (≈ 970 kDa), human serum albumin (HSA ≈ 66 kDa), and bovine IgG (bIgG ≈ 150 kDa), each tested at 5 μg mL^−1^ in HBS-EP buffer. In agreement with previous results obtained for MIPNE in the nanofilm format [20, 23], the biosensor exhibited a markedly higher response to hIgG1 compared to all tested interferents (Fig. 6B), resulting in high selectivity factors (α). This provides evidence that specificity in MIPNE is primarily dictated by the imprinted epitope, rather than by the material format or preparation route. MIPNE-NPs immobilized by A-FM approach were subsequently evaluated for quantitative hIgG1 detection using multi-cycle kinetic (MCK) in the HBS-EP buffer (Fig. 6C). From the calibration, LOD and LOQ values of 0.26 ± 0.05 nmol L^−1^ and 0.63 ± 0.05 nmol L^−1^ were obtained, respectively, with an RSD of 9%. The calculated K_D_ and R_max_ were (1.9 ± 0.7) × 10^−8^ mol L^−1^ and 580 RU, respectively. Finally, the quantitative performance of the biosensor was evaluated in human serum. The sub-nanomolar sensitivity achieved in the buffer, together with the high selectivity, enabled extensive matrix dilution (1:2000) to ensure measurements within the linear range of the sensor. To better determine LOD and LOQ in diluted serum as a blank, endogenous hIgG1 was first depleted by ultrafiltration using a 100 kDa molecular weight cut-off. The resulting depleted serum was then employed as a representative complex matrix for calibration by standard addition of hIgG1. This combined approach enabled decoupling analytical sensitivity from specificity evaluation while preserving biological relevance, reaching LOD = 0.23 ± 0.09 nmol L^−1^ and LOQ = 4.1 ± 0.9 nmol L^−1^, with an RSD of 3%. These values are well below the physiological concentration of hIgG1 in human serum [33], confirming that the biosensor is sufficiently sensitive for clinical-relevant measurements while allowing robust operation in complex biological matrices. Notably, the near-complete overlap between calibration curves obtained in buffer and diluted serum (Fig. 6C), together with identical kinetic parameters observed in both matrices (K_D_ = (1.8 ± 0.2) × 10^−8^ mol L^−1^ and R_max_ = 650 RU; Table S9), further confirms the high specificity and consistent binding performance of the MIPNE receptor. Consistently, recovery experiments in serum showed values of 95 ± 6%, confirming the accuracy and reliability of the biosensor in biologically relevant samples. In contrast to several recent reports on highly sensitive MIP-based IgG sensors, often achieving LODs in the ng mL^−1^ or even pg mL^−1^ range but typically validated only in buffer systems [34–38], the present study provides a systematic assessment of analytical performance directly in serum. For comparison, solid-phase–synthesized rabbit IgG-imprinted nanoMIPs have been reported with K_D_ values of 10–20 × 10^−8^ mol L^−1^ (K_eq_ ≈ 10^6^–10^7^ L mol^−1^), as determined by equilibrium partition experiments in phosphate buffer [38, 39]. However, these studies were restricted to ideal buffer conditions and did not include validation in complex biological matrices, nor the determination of LOD and LOQ, thereby limiting their practical relevance. Overall, the results demonstrate that MIPNE-NPs preserve both high affinity and specificity under physiologically relevant conditions, establishing the present work as a more comprehensive and application-oriented benchmark for MIP-based IgG recognition systems and underscoring their potential for reliable bioanalytical applications in complex real-world samples.

### Sensor reconditioning and reusability

Independently from the specific epitope imprinted, the A-FM strategy provides a general and major advantage over CT-FM and planar nanofilms by enabling a fully in-flow, regenerable workflow. Although total preparation time for MIPNE-NPs (≈24 h) is similar to nanofilms, a single NPs batch can be stored up to months and then reused while retaining the analytical performance (data not shown), avoiding repeated synthesis for each assay or measurement session. This modularity, combined with A-FM’s full automation, reduces operator effort and speeds up assay turnaround. Unlike nanofilms and CT-FM, which require on-chip polymerization or surface preparation, A-FM integrates surface modification, NPs immobilization, and reconditioning entirely under flow. Moreover, MIPNE-NPs can be removed in situ by 3 sequential injections of 0.1 mol L^−1^ NaClO (120 s each) [26], followed by baseline stabilization, fully restoring the gold surface without manual handling. Repeatability was confirmed by 3 immobilization-SCK-removal cycles with H_2_O/NaOH-derived MIPNE-NPs (200 μmol L^−1^ peptide), showing consistent binding, efficient reconditioning, and stable baseline recovery (Table S10-S12, Fig. S6). Inter-cycle repeatability was assessed, with coefficient of variation (CV) values of 4.0% for the epitope and 9.9% for full-length hIgG1, calculated as the average across all tested concentrations (Fig. 6D, Fig. S7). Batch-to-batch synthesis reproducibility was confirmed across independent NP preparations (*n* = 8), which produced particles with consistent size distributions (RSD = 7%). Finally, the ability to store and deploy different batches of MIPNE-NPs, each imprinted for a specific analyte, opens the possibility to perform multi-target detection simply by switching NPs formulations in flow, without replacing the SPR chip, thus saving not only time but also the intrinsic value of the gold sensor surface.

## Conclusions

This work advances the development of MIPNE-NPs as bioinspired synthetic receptors by demonstrating their selective recognition of human IgG1 as a model protein through rational epitope selection and systematic process optimization. The optimized MIPNE-NPs exhibited high binding affinity (K_D_ = (1.9 ± 0.7) × 10^−8^ mol L^−1^) and a binding capacity up to R_max_ ≈ 580 RU, markedly outperforming the corresponding MIPNE nanofilm format. Quantitative evaluation in serum further confirmed the selectivity of the recognition process, with LOD and LOQ values of 0.5 and 0.66 mg mL^−1^, respectively, enabling extensive matrix dilution while preserving analytical performance. Beyond analytical metrics, a key contribution of this study lies in the methodological refinement of MIPNE-NP processing, including the introduction of a fully aqueous and green purification protocol compatible with biologically relevant environments. The comparative assessment of synthetic routes and immobilization strategies demonstrated that formulation parameters and surface chemistry critically influence recognition behavior. Notably, adsorption-based immobilization in flow mode (A-FM) yielded high-affinity binding while enabling complete reconditioning of the sensing interface, supporting sensor reusability and operational continuity. Overall, the combination of affinity, selectivity, biocompatibility, and regenerability positions MIPNE-NPs as versatile synthetic receptors for biosensing applications. While the present study focuses on IgG1 as a representative target, the proposed design and immobilization strategies provide a transferable framework for the development of reusable MIP-based receptors toward other biologically relevant analytes.

## Supplementary information

ESM 1 (DOCX 5.76 MB)

## Data Availability

No datasets were generated or analysed during the current study.
