# Supplementary material for: Biocompatible molecularly imprinted polynorepinephrine nanoparticles: rational design and one-step reversible immobilization for enhanced protein recognition by surface plasmon resonance
Source: Mikrochim Acta. 2026 Feb 19;193(3):171. doi: 10.1007/s00604-026-07893-z (PMC12920386; doi:10.1007/s00604-026-07893-z)
Supplement: Supplementary file 1 — (DOCX 5.76 MB) [file 604_2026_7893_MOESM1_ESM.docx]

**Biocompatible Molecularly Imprinted Polynorepinephrine Nanoparticles: Rational Design and One-Step Reversible Immobilization for Enhanced Protein Recognition by SPR**

S. Ventisette^a^, G. Galgani^b^, P. Palladino^a^, V. Calderone^b^, V. Citi^b^, M. Minunni^b*^, and S. Scarano^a*^

^a^Department of Chemistry “Ugo Schiff’, University of Florence, Via della Lastruccia, 3-13, 50019 Sesto Fiorentino, Italy

^b^Department of Pharmacy, University of Pisa, Via Bonanno 6, 56126, Pisa, Italy

*Corresponding authors: simona.scarano@unifi.it, maria.minunni@unipi.it

**S1. Chemicals and reagents**

L-norepinephrine hydrochloride (NE, ≧ 98.0%), tris(hydroxymethyl)aminomethane hydrochloride (TRIS-HCl, ≧ 99.0%), 4-(2-Hydroxyethyl) piperazine-1-ethanesulfonic acid (HEPES), ethylenediaminetetraacetic acid disodium salt solution (EDTA-Na_2_), sodium dodecyl sulfate (SDS), sodium chloride, sodium hydroxide, sodium hypochlorite, hydrochloric acid, potassium chloride, di-sodium hydrogen phosphate dihydrate, sodium dihydrogen phosphate monohydrate, acetonitrile (ACN ≧ 99.5%), 6-mercapto-1-hexanol (MCH, 97%), 11-mercapto-1-undecanol (MCU, 97%), 1,4-benzenedimethanthiol (BDMT), ethanol, polyoxyethylene sorbitan monooleate (Tween-20), human serum albumin (HSA), IgG from bovine serum (bIgG), Dulbecco’s modified eagle’s medium - high glucose (DMEM-HG), fetal bovine serum (FBS), 1% penicillin-streptomycin (P/S, 10,000 U mL^-1^ penicillin and 10 mg mL^-1^ streptomycin), Dulbecco’s Phosphate Buffered Saline, water soluble tetrazolium - 1 (WST-1), and sterile-filtered HS (from human male AB plasma) were purchased from Merck (Darmstadt, Germany). Peptide sequences ^441^KSLSLSPGK^449^ (MW = 916.08 g mol^−1^) and ^338^ISKAKGQP^345^ (MW = 827.98 g mol^−1^), belonging to the heavy chain of the immunoglobulins’ constant domain, were provided by GenScript (Leiden, Netherlands) with an HPLC purity above 95.0%. Human IgA (catalog #31148), IgM (catalog #31146), IgE (catalog #DIA HE1-01), and IgG (catalog #02–7102) isotype controls were purchased from Thermo Fisher Scientific (Monza, Italy). Human immortalized keratinocytes (HaCaT) cells were purchased from ATCC (Manassas, Virginia, USA). Ultrapure Milli-Q^TM^ water (R ≧ 18.2 MΩ cm) was used for the preparation of buffer solutions. HEPES-buffered saline (HBS-EP: 10 mmol L^−1^ HEPES, 150 mmol L^−1^ NaCl, 3 mmol L^−1^ EDTA, 0.005% Tween-20, pH 7.4) and phosphate-buffered saline (PBS: 140 mmol L^−1^ NaCl, 2.68 mmol L^−1^ KCl, 3.56 mmol L^−1^ NaH_2_PO_4_, 6.44 mmol L^−1^ Na_2_HPO_4_, pH 7.4), filtered through a 0.22 µm Millipore^TM^ microporous filter (Merck, Darmstadt, Germany), were used as buffer for all the SPR analysis. Amicon^®^ Ultra Centrifugal 0.5 mL filters with molecular weight cut-off of 100 kDa (Merck, Darmstadt, Germany), were used to remove H-IgG1 from human serum. All chemicals used were of analytical grade.

**S2. Scanning Electron Microscopy (SEM) and Scanning Transmission Electron Microscopy (STEM)**

SEM analyses were performed using a Phenom Pharos G2 Desktop Field Emission Gun Scanning Electron Microscope (FEG-SEM) (Thermo Fisher Scientific, Waltham, MA, USA). Aqueous solutions (4 µL each) of MIPNE-NPs were drop-casted onto a silica wafer and allowed to air dry. Subsequently, the samples were coated with a conductive graphite layer using an EM ACE200 coating system (Leica Microsystems, Wetzlar, Germany), applying approximately 3 nm of graphite in 10 pulses. SEM images were acquired under high vacuum conditions (0.10 Pa) with an acceleration voltage of 20 kV, employing the Secondary Electron Detector (SED). STEM analyses were conducted using the STEM detector equipped on the SEM instrument. Aqueous solutions (4 µL each) of MIPNE-NPs were deposited onto TEM Formvar-Carbon supported copper grids (200 square mesh) and allowed to dry. STEM measurements were carried out under high vacuum conditions (0.10 Pa) with an acceleration voltage of 15 kV. Nanoparticle size analyses were performed using Phenom Pro Suite particlemetric software (Thermo Fisher Scientific, Waltham, MA, USA) to measure the diameters of at least 200 NPs from representative SEM and STEM images.

**Table S1** Experimental variables and their respective levels (low and high) employed in the DoE screening for both H_2_O/NaOH and TRIS (10 mmol L^-1^) conditions.

|  | **H_2_O/NaOH** | | **10 mmol L^-1^**  **TRIS buffer** | |
| --- | --- | --- | --- | --- |
| **Variable** | **Lower value** | **Higher value** | **Lower value** | **Higher value** |
| **pH** | 11 | 13 | 7 | 11 |
| **T (°C)** | 30 | 70 | 30 | 70 |
| **t (h)** | 2 | 8 | 2 | 8 |
| **Stirring (RPM)** | 400 | 1000 | 400 | 1000 |
| **NE concentration (g L^-1^)** | 1 | 5 | 1 | 5 |

**Table S2** H_2_O/NaOH DoE experimental plan

| **#A** | **pH** | **T (°C)** | **t (h)** | **Stirring (RPM)** | **NE conc. (g L^-1^)** |
| --- | --- | --- | --- | --- | --- |
| 1_NaOH | 13 | 30 | 2 | 1000 | 5 |
| 2_NaOH | 13 | 70 | 8 | 1000 | 5 |
| 3_NaOH | 11 | 70 | 2 | 1000 | 1 |
| 4_NaOH | 13 | 70 | 8 | 400 | 1 |
| 5_NaOH | 13 | 30 | 2 | 400 | 1 |
| 6_NaOH | 12 | 50 | 5 | 700 | 3 |
| 7_NaOH | 11 | 70 | 2 | 400 | 5 |
| 8_NaOH | 12 | 50 | 5 | 700 | 3 |
| 9_NaOH | 11 | 30 | 8 | 400 | 5 |
| 10_NaOH | 11 | 30 | 8 | 1000 | 1 |
| 11_NaOH | 12 | 50 | 5 | 700 | 3 |

**Table S3** 10 mmol L^-1^ TRIS buffer DoE experimental plan

| **#B** | **pH** | **T (°C)** | **t (h)** | **Stirring (RPM)** | **NE conc. (g L^-1^)** |
| --- | --- | --- | --- | --- | --- |
| 1_TRIS | 7 | 30 | 8 | 1000 | 1 |
| 2_TRIS | 7 | 30 | 8 | 400 | 5 |
| 3_TRIS | 10 | 70 | 8 | 400 | 1 |
| 4_TRIS | 7 | 70 | 2 | 1000 | 1 |
| 5_TRIS | 8.5 | 50 | 5 | 700 | 3 |
| 6_TRIS | 8.5 | 50 | 5 | 700 | 3 |
| 7_TRIS | 10 | 30 | 2 | 1000 | 5 |
| 8_TRIS | 10 | 70 | 8 | 1000 | 5 |
| 9_TRIS | 10 | 30 | 2 | 400 | 1 |
| 10_TRIS | 7 | 70 | 2 | 400 | 5 |
| 11_TRIS | 8.5 | 50 | 5 | 700 | 3 |


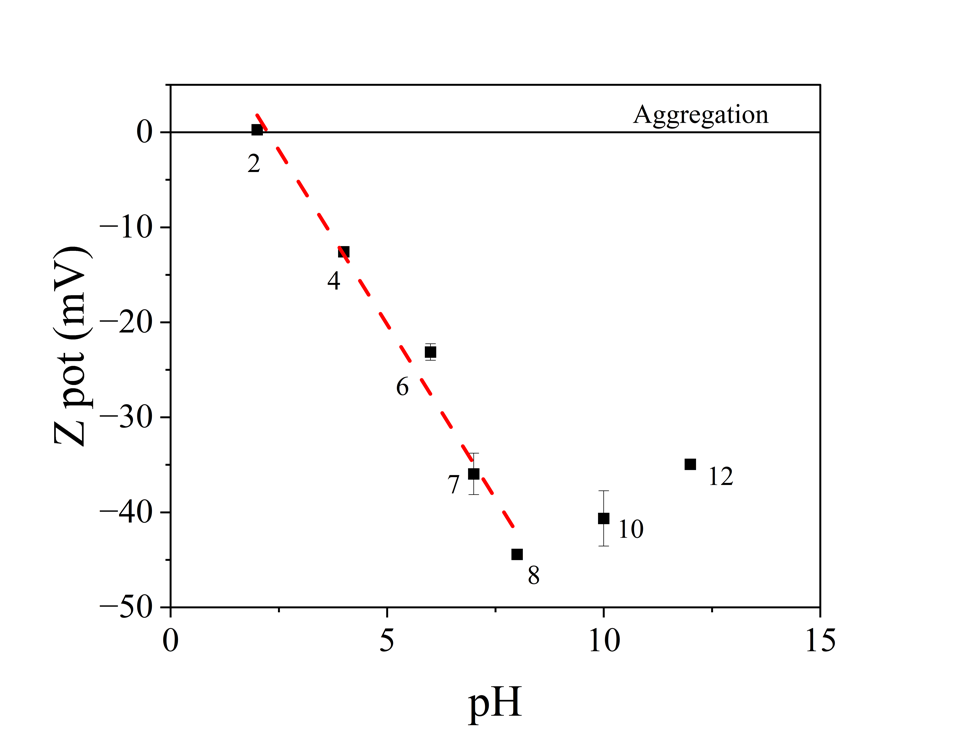


**Fig. S1** Dependence of MIPNE‑NP zeta potential values on solution pH.

**Table S4** H_2_O/NaOH and 10 mmol L^-1^ TRIS NIPNE-NPs characterization

| **NaOH#** | **DLS diameter (nm)** | **PDI** | **Abs@700 nm (a.u.)** |  | **TRIS#** | **DLS diameter (nm)** | **PDI** | **Abs@700 nm (a.u.)** |
| --- | --- | --- | --- | --- | --- | --- | --- | --- |
| 1_NaOH | 324 ± 5 | 0.22 ± 0.01 | 0.0348 ± 0.0006 |  | 1_TRIS | / | / | / |
| 2_NaOH | 125.9 ± 0.7 | 0.16 ± 0.03 | 0.128 ± 0.001 |  | 2_TRIS | / | / | / |
| 3_NaOH | 180.50 ± 0.07 | 0.26 ± 0.01 | 0.05 ± 0.08 |  | 3_TRIS | 170 ± 1 | 0.23 ± 0.01 | 0.075 ± 0.008 |
| 4_NaOH | 189 ± 4 | 0.32 ± 0.02 | 0.0038 ± 0.0006 |  | 4_TRIS | 185 ± 1 | 0.31 ± 0.03 | 0.014 ± 0.001 |
| 5_NaOH | 297 ± 15 | 0.28 ± 0.03 | 0.0022 ± 0.0006 |  | 5_TRIS | 277 ± 5 | 0.21 ± 0.01 | 0.255 ± 0.008 |
| 6_NaOH | 273 ± 7 | 0.22 ± 0.02 | 0.194 ± 0.004 |  | 6_TRIS | 227 ± 4 | 0.22 ± 0.02 | 0.098 ± 0.002 |
| 7_NaOH | 721 ± 61 | 0.38 ± 0.06 | 0.037 ± 0.002 |  | 7_TRIS | 577 ± 17 | 0.38 ± 0.02 | 0.05 ± 0.06 |
| 8_NaOH | 246 ± 6 | 0.23 ± 0.01 | 0.18 ± 0.01 |  | 8_TRIS | 294 ± 3 | 0.195 ± 0.008 | 1.095 ± 0.003 |
| 9_NaOH | 627 ± 90 | 0.51 ± 0.01 | 0.016 ± 0.002 |  | 9_TRIS | 422 ± 24 | 0.5 ± 0.1 | 0.001 ± 0.001 |
| 10_NaOH | 180.00 ± 0.02 | 0.212 ± 0.007 | 0.05 ± 0.01 |  | 10_TRIS | 700 ± 56 | 0.56 ± 0.07 | 0.069 ± 0.001 |
| 11_NaOH | 238 ± 2 | 0.2387 ± 0.0009 | 0.171 ± 0.005 |  | 11_TRIS | 284 ± 5 | 0.20 ± 0.02 | 0.181 ± 0.002 |

**Fig. S2** NIPNE-NPs’ cytotoxicity on HaCaT keratinocytes. Cells were exposed to different NIPNE-NPs (optimized 3_NaOH, 2_NaOH, 8_NaOH, 3_TRIS, and 8_TRIS), each applied at concentrations of 10, 30, and 100 ng/mL, for 24 h. Data represent mean SPR response (n=6) ± SD. Statistical analysis was performed using one-way ANOVA followed by Bonferroni post hoc tests; no significant differences compared to untreated controls were detected (p > 0.05).

**S3. Effect of the hIgG1 epitope concentration on PNE-NPs imprinting**

Building on the optimized NIPNE‑NPs protocols, the epitope template ^441^KSLSLSPGK^449^, designed to bind hIgG1, was introduced to obtain MIPNE-NPs. The DoE optimization was deliberately restricted to the non-imprinted system in order to decouple the fundamental parameters governing nanoparticle formation (size, colloidal stability, and surface chemistry) from the additional effects introduced by the template. Subsequently, the epitope concentration was tuned outside the DoE framework to specifically isolate the direct contribution of peptide-induced cross-linking and imprinting on the polymer network, avoiding confounding interactions among multiple variables and preserving mechanistic interpretability. As recently reported [1], the increase in MIPNE film thickness correlates with the peptide’s ability to cross-link the polymer matrix during the imprinting process. Accordingly, the MIPNE absorbance is directly related to the peptide’s “imprint-ability”, which, in turn, is determined by its primary amino acid sequence and physicochemical interactions with polymerizing PNE. The peptide was added ranging from 50 to 400 µmol L^-1^, at the start of polymerization in both media. DLS measurements confirmed D_h_ values below 200 nm across all conditions, though a peptide concentration-dependent increase was observed (Fig. S3). In H_2_O/NaOH, D_h_ rose from 180 to 191 nm, whereas in TRIS this dependence appears more pronounced (from 170 to 199 nm), suggesting a greater tendency of PNE-NPs to incorporate peptides under these conditions. This difference can be rationalized by considering the chemical role of the reaction medium on NE oxidation and polymer growth. Under alkaline conditions, NE undergoes catechol oxidation to quinone intermediates, followed by oligomerization and cross-linking through Michael-type reactions [2]. TRIS, owing to the presence of a primary amine, can act as a nucleophilic quencher of quinone species, forming Schiff base or Michael-type adducts with oxidized catechol units [2]. These competing reactions partially cap reactive sites, effectively moderating polymer growth and slowing network closure. Such kinetic control is expected to favor the incorporation of the imprinting epitope into the growing polymer network. Conversely, in the H_2_O/NaOH system, the absence of nucleophilic buffer components results in faster, less regulated oxidation and polymerization, promoting rapid network formation and limiting effective template integration. Overall, these findings highlight how buffer identity influences oxidation kinetics, oligomerization pathways, and ultimately imprinting efficiency in PNE-based nanoparticle systems.

**S4. Effect of hIgG1 epitope concentration on binding efficiency**

MIPNE-NPs synthesized with different initial epitope concentrations (paragraph 3.4) were immobilized and compared in terms of peptide-binding kinetics. For H_2_O/NaOH-derived NPs, binding capacity (R_max_) increased with epitope concentration (28-91 RU), while K_D_ followed a non-linear trend (Fig. S5, Table S6). Conversely, TRIS-derived NPs displayed overall lower binding capacities (26-44 RU) but consistently higher affinities (Table S7). Comparing the two synthesis methods, H_2_O/NaOH-based NPs provided higher binding capacity, whereas TRIS particles offered stronger affinity, particularly at low epitope concentrations. The optimal compromise was H_2_O/NaOH synthesis at 200 µmol L^-1^, yielding both low K_D_ (4.30×10^-10^ mol L^-1^) and high R_max_ (80 RU).


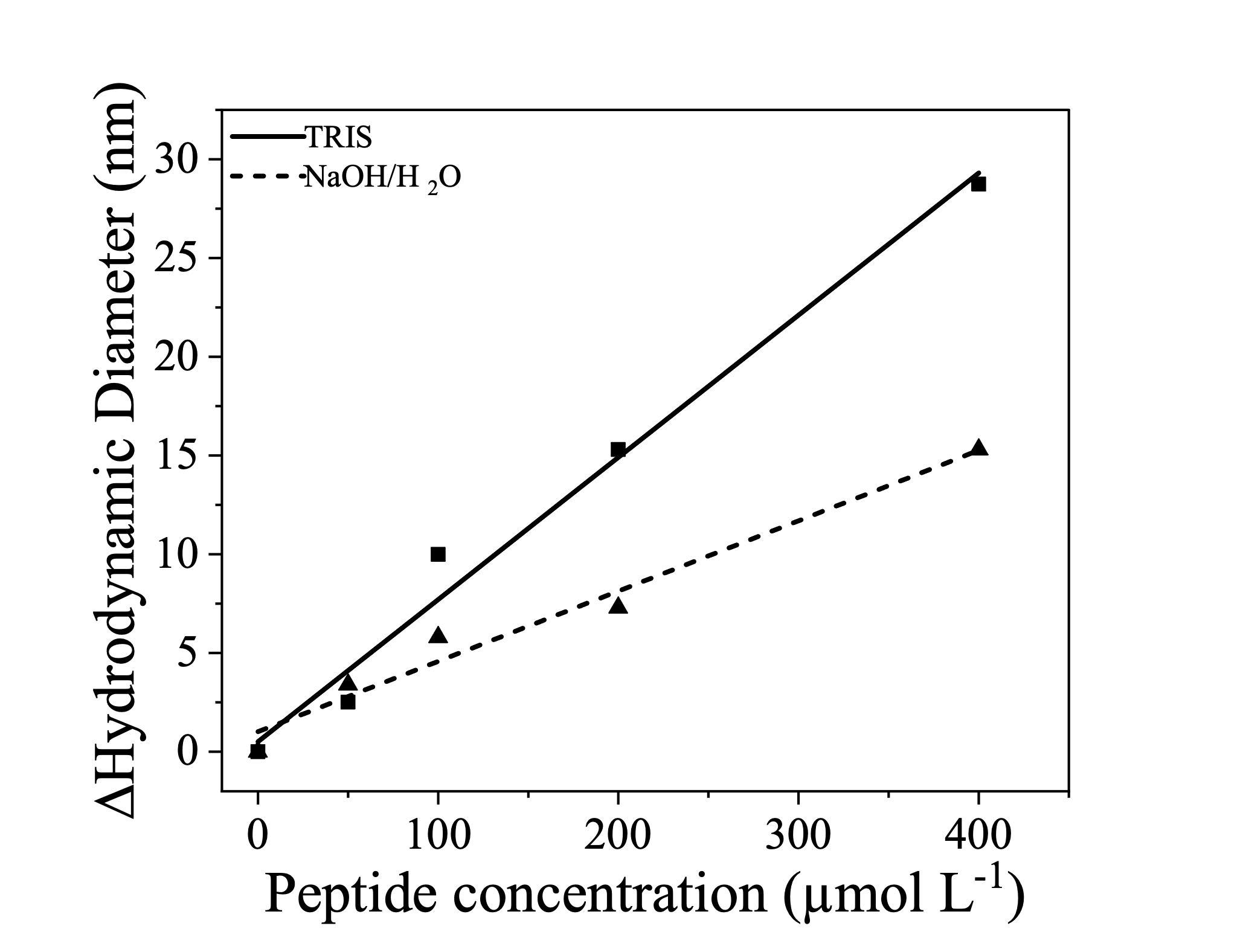


**Fig. S3** Dependence of MIPNE‑NPs diameter on IgG1 peptide template concentration in H_2_O/NaOH and TRIS buffer. D_h_ are measured by DLS as a function of added peptide concentration for syntheses in H_2_O/NaOH (triangles, dashed line) and TRIS (squares, solid line).


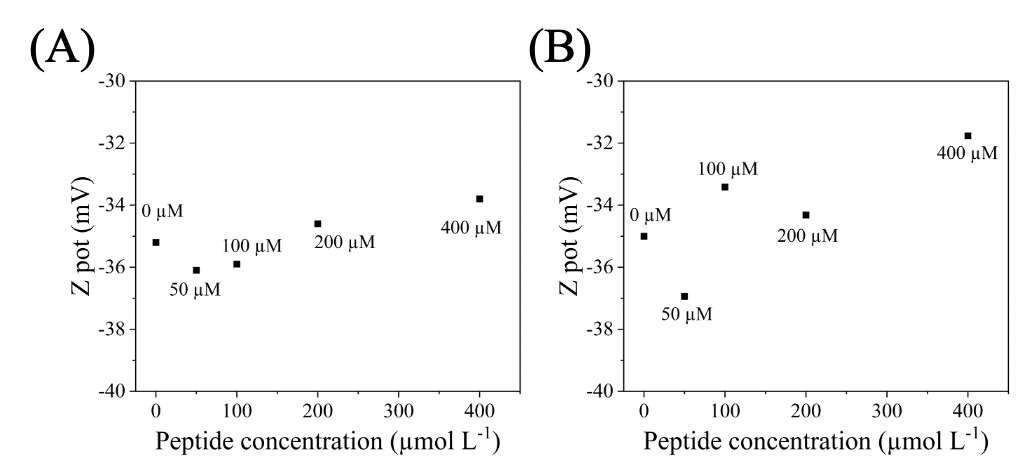


**Fig. S4** Dependence of MIPNE‑NP zeta potential values on hIgG1 peptide template concentration in (A) H_2_O/NaOH and (B) TRIS buffers.


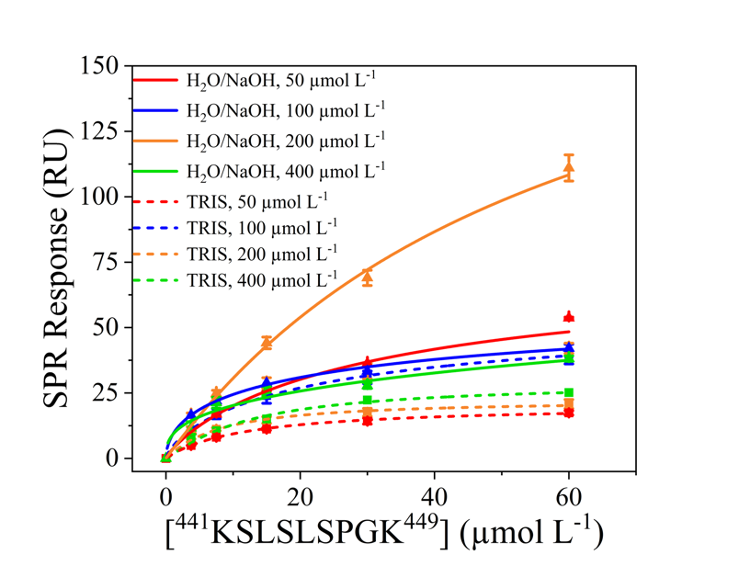


**Fig. S5** Comparison of MIPNE‐NPs immobilized via A-FM, obtained in H_2_O/NaOH or TRIS solution using different template concentrations

**Table S5** SCK peptide analysis for immobilization methods comparison.

| **Synthesis medium** | **Immob. method** | **k_a1_**  **(mol^-1^ L s^-1^)** | **k_d1_**  **(s-^1^)** | **k_a2_**  **(s^-1^)** | **k_d2_**  **(s^-1^)** | **K_D_**  **(mol L^-1^)** | **R_max_**  **(RU)** |
| --- | --- | --- | --- | --- | --- | --- | --- |
| **TRIS** | **CT** | 172700 | 0.03962 | 1.38 • 10^-3^ | 1.35 • 10^-3^ | 1.17 • 10^-5^ | 35 |
|  | **A** | 4349 | 0.1303 | 4.91 • 10^-3^ | 1.91 • 10^-6^ | 1.16 • 10^-8^ | 34 |
| **NaOH** | **CT** | 4246 | 0.1131 | 5.47 • 10^-3^ | 1.47 • 10^-3^ | 5.65 • 10^-6^ | 80 |
|  | **A** | 444500 | 2.355 | 2.84 • 10^-3^ | 2.31 • 10^-7^ | 4.30 • 10^-10^ | 80 |

**Table S6** Analytical parameters for H_2_O/NaOH MIPNE-NPs, SCK peptide analysis.

| **Peptide concentration (µmol L^-1^)** | **k_a1_**  **(mol^-1^ L s^-1^)** | **k_d1_**  **(s-^1^)** | **k_a2_**  **(s^-1^)** | **k_d2_**  **(s^-1^)** | **K_D_**  **(mol L^-1^)** | **R_max_**  **(RU)** |
| --- | --- | --- | --- | --- | --- | --- |
| 50 | 26610 | 0.2475 | 3.74 • 10^-3^ | 1.77 • 10^-4^ | 4.20 • 10^-7^ | 28 |
| 100 | 15930 | 0.1686 | 2.43 • 10^-3^ | 1.65 • 10^-3^ | 4.28 • 10^-6^ | 60 |
| 200 | 444500 | 2.355 | 2.84 • 10^-3^ | 2.31 • 10^-7^ | 4.30 • 10^-10^ | 80 |
| 400 | 9467 | 0.1686 | 3.23 • 10^-4^ | 2.71 • 10^-6^ | 1.48 • 10^-7^ | 91 |

**Table S7** Analytical parameters for TRIS MIPNE-NPs, SCK peptide analysis.

| **Peptide concentration (µmol L^-1^)** | **k_a1_**  **(mol^-1^ L s^-1^)** | **k_d1_**  **(s-^1^)** | **k_a2_**  **(s^-1^)** | **k_d2_**  **(s^-1^)** | **K_D_**  **(mol L^-1^)** | **R_max_**  **(RU)** |
| --- | --- | --- | --- | --- | --- | --- |
| 50 | 22380 | 0.24 | 1.96 • 10^-3^ | 3.96 • 10^-6^ | 2.16 • 10^-8^ | 26 |
| 100 | 10550 | 0.1447 | 4.03 • 10^-3^ | 1.17 • 10^-6^ | 3.96 • 10^-9^ | 44 |
| 200 | 9565 | 0.1197 | 2.43 • 10^-3^ | 7.67 • 10^-7^ | 3.95 • 10^-9^ | 27 |
| 400 | 4349 | 0.1303 | 4.91 • 10^-3^ | 1.91 • 10^-6^ | 1.16 • 10^-8^ | 34 |

**Table S8** Analytical parameters for H_2_O/NaOH (200 µmol L^-1^ peptide) MIPNE-NPs, SCK protein analysis.

|  | **k_a_**  **(mol^-1^ L s^-1^)** | **k_d_**  **(s^-1^)** | **K_D_**  **(mol L^-1^)** | **R_max_**  **(RU)** |
| --- | --- | --- | --- | --- |
| **CT-FM** | (5.6420 ± 0.0005) • 10^7^ | (1.3 ± 0.1) • 10^-2^ | (2.23 ± 0.02) • 10^-10^ | (4.3 ± 0.3) • 10^3^ |
| **A-FM** | (4.16 ± 0.05) • 10^4^ | (6.38 ± 0.09) • 10^-4^ | (1.53 ± 0.03) • 10^-8^ | 844 ± 8 |
| **film** | (2.05 ± 0.04) • 10^2^ | (9.83 ± 0.06) • 10^-3^ | (4.8 ± 0.1) • 10^-5^ | 108 ± 1 |

**Table S9** Analytical parameters for H_2_O/NaOH (200 µmol L^-1^ peptide) MIPNE-NPs, MCK protein analysis.

|  | **k_a_**  **(mol^-1^ L s^-1^)** | **k_d_**  **(s^-1^)** | **R_max_**  **(RU)** | **K_D_**  **(mol L^-1^)** | **RSD**  **%** | **LOD (nmol L^-1^)** | **LOQ (nmol L^-1^)** | **CV %** |
| --- | --- | --- | --- | --- | --- | --- | --- | --- |
| **A-FM (buffer)** | 1.5 ± 0.5 ·10^5^ | 2.9 ± 0.5 ·10^-3^ | 5.8 ± 0.5 ·10^2^ | 1.9 ± 0.7 ·10^-8^ | 34 | 0.26 ± 0.05 | 0.63 ± 0.5 | 9 |
| **A-FM (1/2000 serum)** | 1.6 ± 0.2 ·10^5^ | 2.8 ± 0.1 ·10^-3^ | 6.5 ± 0.2 ·10^2^ | 1.8 ± 0.2 ·10^-8^ | 11 | 0.23 ± 0.09 | 4.1 ± 0.9 | 3 |


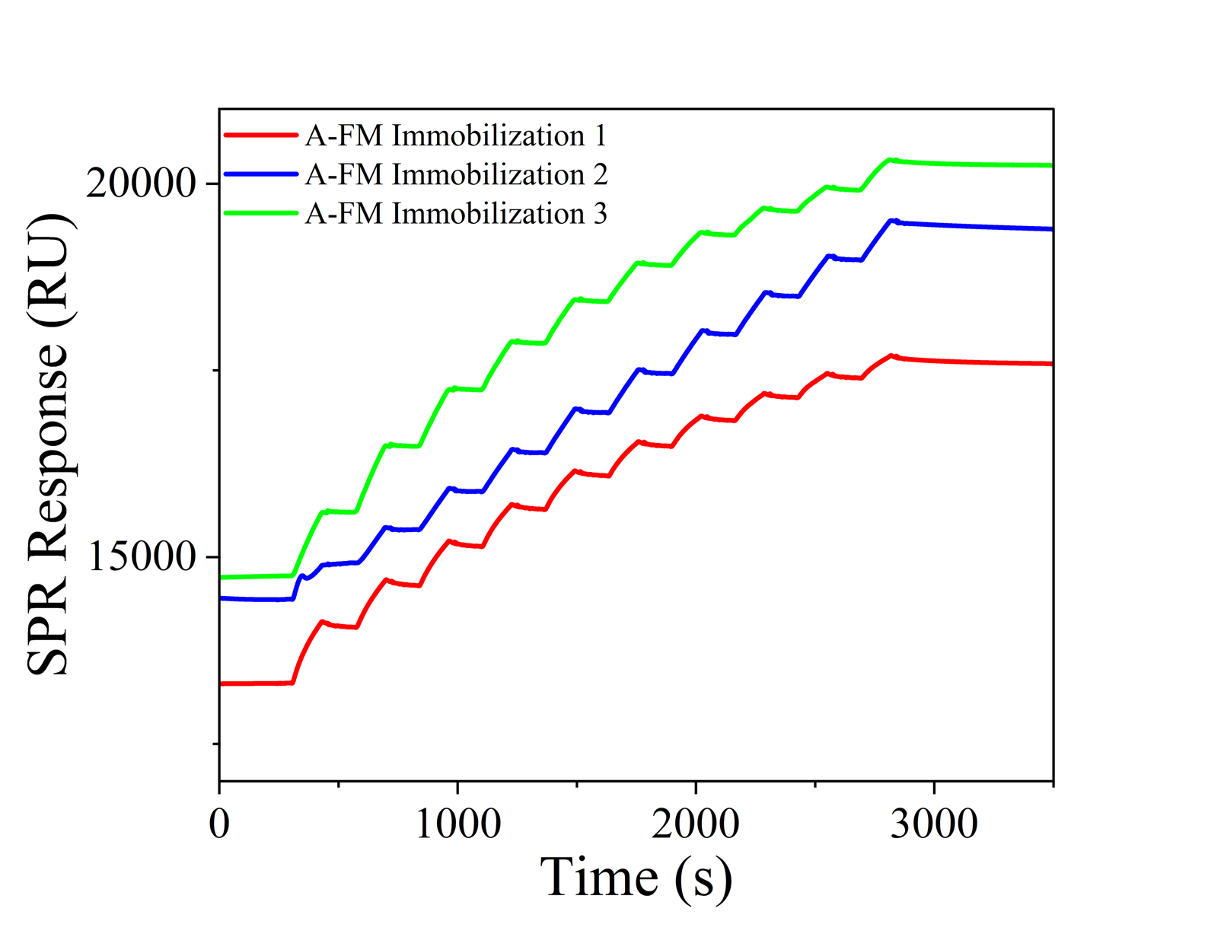


**Fig. S6** Immobilization levels obtained across three successive immobilization rounds of H_2_O/NaOH MIPNE-NPs (200 µmol L^-1^ peptide) via the A-FM.


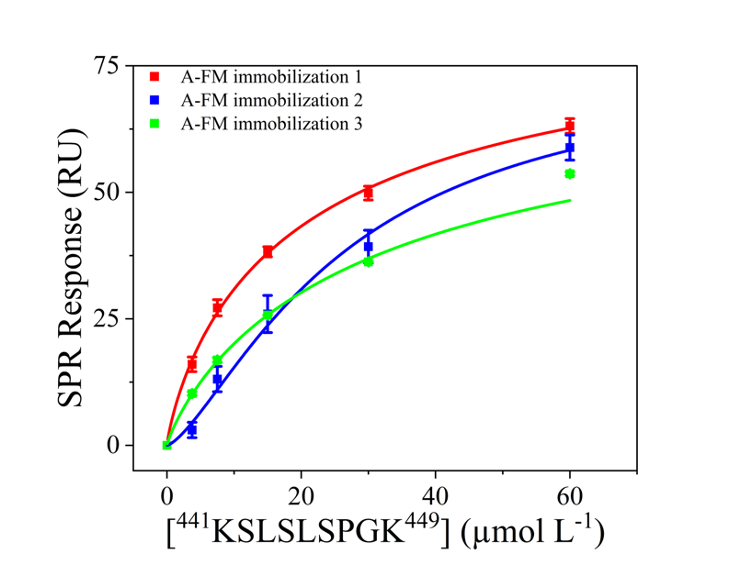


**Fig. S7** Accumulation points from SCK analysis of peptide ^441^KSLSLSPGK^449^ over three consecutive immobilization cycles via A-FM.

**Table S10** SPR responses recorded during three successive MIPNE-NPs depositions by NPs-AAuFM (each deposition comprising 10 sequential 120 s injections) and subsequent in-flow removal (three sequential 120 s injections of 0.1 mol L^-1^ NaOCl).

|  | **Baseline (RU)** | **Stability after 10 injections (RU)** | **Net signal gain (RU)** | **Baseline (RU)** | **Stability after 3 injections (RU)** | **Net signal loss (RU)** |
| --- | --- | --- | --- | --- | --- | --- |
| NPs-AAuFM Deposition 1 | 13315.1 | 17691.2 | 4376.1 | 18144.3 | 14699.6 | -3445 |
| NPs-AAuFM Deposition 2 | 14439.1 | 19503.4 | 5064.3 | 21043.6 | 14790.7 | -6253 |
| NPs-AAuFM Deposition 3 | 14748.9 | 20312.4 | 5563.5 | 20750.8 | 14721.8 | -6029 |
| Average | (14.2 ± 0.8) • 10^3^ | (19 ± 1) • 10^3^ | (5.0 ± 0.6) • 10^3^ | (20 ± 2) • 10^3^ | (14.74 ± 0.05) • 10^3^ | (5 ± 2) • 10^3^ |

**Table S11** Analytical parameters recorded during three successive immobilization/removal of H_2_O/NaOH (200 µmol L^-1^ peptide) MIPNE-NPs, SCK peptide analysis.

|  | **k_a1_**  **(mol^-1^ L s^-1^)** | **k_d1_**  **(s-^1^)** | **k_a2_**  **(s^-1^)** | **k_d2_**  **(s^-1^)** | **K_D_**  **(mol L^-1^)** | **R_max_**  **(RU)** |
| --- | --- | --- | --- | --- | --- | --- |
| NPs-AAuFM deposition 1 | 444500 | 2.355 | 2.84 • 10^-3^ | 2.31 • 10^-7^ | 4.30 • 10^-10^ | 80 |
| NPs-AAuFM deposition 2 | 2126 | 0.1545 | 1.01 • 10^-2^ | 1.59 • 10^-6^ | 1.15 • 10^-8^ | 68 |
| NPs-AAuFM deposition 3 | 26610 | 0.2475 | 3.74 • 10^-3^ | 1.77 • 10^-4^ | 4.20 • 10^-7^ | 28 |

**Table S12** Kinetic parameters inferred from three successive immobilization/removal of H_2_O/NaOH (200 µmol L^-1^) MIPNE-NPs, SCK protein analysis.

|  | **k_a_**  **(mol^-1^ L s^-1^)** | **k_d_**  **(s^-1^)** | **K_D_**  **(mol L^-1^)** | **R_max_**  **(RU)** |
| --- | --- | --- | --- | --- |
| NPs-AAuFM deposition 1 | (2.7 ± 0.7) • 10^4^ | (1.9 ± 0.4) • 10^-2^ | (6.9 ± 0.4) • 10^-7^ | (1.4 ± 0.2) • 10^4^ |
| NPs-AAuFM deposition 2 | (5.37 ± 0.04) • 10^4^ | (6.06 ± 0.09) • 10^-4^ | (1.13 ± 0.02) • 10^-8^ | 667 ± 5 |
| NPs-AAuFM deposition 3 | (4.16 ± 0.05) • 10^4^ | (6.38 ± 0.09) • 10^-4^ | (1.53 ± 0.03) • 10^-8^ | 844 ± 8 |

**References**

1. Sestaioni D, Giurlani W, Ciacci G, et al (2024) Sustainable and effective reconditioning of SPR gold chips functionalized with molecularly imprinted polynorepinephrine. Anal Chim Acta 1321:343037. https://doi.org/10.1016/j.aca.2024.343037

2. Ryu JH, Messersmith PB, Lee H (2018) Polydopamine Surface Chemistry: A Decade of Discovery. ACS Appl Mater Interfaces 10:7523–7540. https://doi.org/10.1021/acsami.7b19865
